# The Function of SUMOylation and Its Critical Roles in Cardiovascular Diseases and Potential Clinical Implications

**DOI:** 10.3390/ijms221910618

**Published:** 2021-09-30

**Authors:** Congcong Du, Xu Chen, Qi Su, Wenbin Lu, Qiqi Wang, Hong Yuan, Zhenzhen Zhang, Xiaotong Wang, Hongmei Wu, Yitao Qi

**Affiliations:** 1 Key Laboratory of the Ministry of Education for Medicinal Resources and Natural Pharmaceutical Chemistry, National Engineering Laboratory for Resource Developing of Endangered Chinese Crude Drugs in Northwest of China, College of Life Sciences, Shaanxi Normal University, Xi’an 710119, China; ducongcong@snnu.edu.cn (C.D.); chenxu@snnu.edu.cn (X.C.); suqidream77@163.com (Q.S.); sdluloloo@163.com (W.L.); wang222703789@snnu.edu.cn (Q.W.); yhong@snnu.edu.cn (H.Y.); zhang2877534304@snnu.edu.cn (Z.Z.); 2 School of Agriculture, Ludong University, Yantai 246011, China; wangxiaotong999@163.com

**Keywords:** SUMOylation, SENP, cardiac development, cardiovascular disease, clinical targets

## Abstract

Cardiovascular disease (CVD) is a common disease caused by many factors, including atherosclerosis, congenital heart disease, heart failure, and ischemic cardiomyopathy. CVD has been regarded as one of the most common diseases and has a severe impact on the life quality of patients. The main features of CVD include high morbidity and mortality, which seriously threaten human health. SUMO proteins covalently conjugate lysine residues with a large number of substrate proteins, and SUMOylation regulates the function of target proteins and participates in cellular activities. Under certain pathological conditions, SUMOylation of proteins related to cardiovascular development and function are greatly changed. Numerous studies have suggested that SUMOylation of substrates plays critical roles in normal cardiovascular development and function. We reviewed the research progress of SUMOylation in cardiovascular development and function, and the regulation of protein SUMOylation may be applied as a potential therapeutic strategy for CVD treatment.

## 1. Introduction

Cardiovascular disease (CVD) is a generic term of heart disease caused by a series of factors and is one of the major causes of human death. CVD is a serious threat to human life and has become a hot topic in modern medical research. SUMOylation acts as a dynamic equilibrium protein modification and is ubiquitous in human cells. It has been found that SUMOylation plays important roles in maintaining normal cell functions, and dysregulation of SUMOylation has been observed in the pathogenesis of various heart diseases. There are a large number of target proteins modified by SUMO in the heart, which affect cell proliferation, apoptosis, and metabolism. The imbalance of SUMOylation leads to abnormal cardiac function, resulting in the occurrence of CVD. However, the molecular mechanism of SUMOylation in CVD is still not completely clear. This review describes the latest research of SUMOylation in CVD and aims to provide new ideas and potential therapeutic targets for the treatment of CVD.

### 1.1. CVD

CVD is a group of diseases caused by many factors, including atherosclerosis, congenital heart disease (CHD), heart failure, and ischemic cardiomyopathy, with a high prevalence, morbidity, and mortality [1]. Atherosclerosis is a progressive inflammatory cardiovascular disease characterized by lipid plaque formation in the artery [2]. The formation and rupture of lipid plaques is one important cause of clinical cardiovascular events, including stroke and myocardial infarction, accounting for more than one-quarter of global deaths [3]. CHD is a congenital cardiac structural abnormality, and it is a common congenital defect in infants, accounting for about 1% of all newborns [4]. Atrial septal defects (ASDs) and ventricular septal defects (VSDs) are the most common CHDs, which are characterized by an existing connection between atria and ventricles [5]. Heart failure is a complex disease caused by valve abnormality, myocardial ischemia, coronary artery disease, hypertension, and other pathological changes [6]. Cardiac energy metabolism and mitochondrial biogenesis defects lead to heart failure and cardiac hypertrophy [7]. Acute myocardial infarction (AMI) is an ischemic cardiac disease with acute hypoxia in the myocardial tissue and is one of the leading causes of death. Ischemia/reperfusion (I/R) is an important treatment for rescuing ischemic myocardium from necrosis [8]. Readjustment of blood flow restores oxygen and nutrient supply but may damage the cardiac muscle and induce myocardial I/R injury. Advances in pharmacology and interventional therapy have improved CVD treatment but have not solved the problem from the source. Extensive proteins involved in the pathology of CVD are modified by SUMO proteins, indicating that SUMOylation of substrates plays important roles in the progression of CVD.

### 1.2. SUMOylation

SUMO (small ubiquitin-like modifier) is ubiquitous in eukaryotes, and the SUMO family contains SUMO1, SUMO2, SUMO3, and SUMO4 [9,10]. SUMO1, SUMO2, and SUMO3 are commonly found [11,12], while SUMO4 is only expressed in the spleen, kidney, and other organs [13]. The identical sequences of SUMO2 and SUMO3 are 97%, so they are difficult to distinguish and commonly referred to as SUMO2/3, while only 47% of the homologous sequences are between SUMO1 and SUMO2/3 [14,15]. SUMO4 was initially shown to be associated with the pathogenesis of type 1 diabetes, but it was later reported that SUMO4 was ineffective in covalent coupling with the substrate due to the presence of proline-90 amino acid residues that prevent maturation of SUMO4 [16].

SUMOylation is accomplished via maturation, activation, conjugation, and ligation with activating enzyme E1 (SAE1/SAE2), conjugating enzyme E2 (UBC9), and ligating enzyme E3 [15]. The C-terminal of SUMO forms a covalent bond with the lysine residues of target proteins through an isopeptide bond. The SUMO precursor is processed to expose the C-terminal di-glycine motif, then the cysteine residue of the E1 subunit forms a high-energy thioester bond with SUMO. The activated SUMO was transferred to the E2-binding enzyme, and linked to the cystine of UBC9 to form the SUMO-E2 intermediate. UBC9 transferred SUMO to the substrate, and the C-terminal glycine of SUMO bound to the lysine residue of the substrate to form the isopeptide bond [17]. Although E1 and E2 are sufficient to complete SUMOylation, the E3 is still required to enhance the specificity of SUMO isoforms and substrates, activate the SUMO binding site, and promote the formation of poly-SUMO chains.

**Figure 1:**
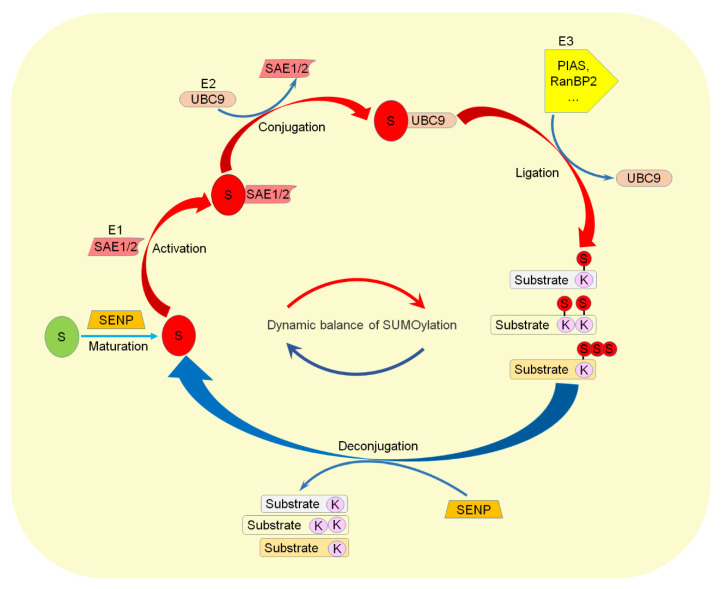
Dynamic regulation of the SUMOylation pathway. The SUMO precursor protein is matured by SENP and then activated by E1, conjugated by E2, and ligated to substrates by E3. On the other hand, SENP deconjugates SUMO from target proteins. S: SUMO. K: Lysine.

The deSUMOylation is achieved by a family of SENP (Sentrin/SUMO-specific protease) [10,18], which deconjugate the SUMO from target proteins and catalyze the maturation of the SUMO proteins [7]. The SENP family in mammalian cells has six members, which can be divided into three groups. The first group is SENP1 and SENP2, and both have broad substrate specificity [19,20,21,22,23,24]. The second group is SENP3 and SENP5, and both in the nucleus, but mainly in the nucleolus, tend to deSUMOylation of SUMO2/3. The third group includes SENP6 and SENP7, both with additional ring structures in their catalytic zone. SUMO and SENP2 dynamically regulate the SUMOylation level of target proteins (Figure 1), and SUMOylation is an important dynamic reversible post-translational modification (PTM) to regulate protein stability, subcellular localization, and function [25]. Disorders of SUMOylation affect the normal function of target proteins and lead to the damage of important physiological processes and an abnormal intracellular environment [26].

Protein homeostasis is indispensable, especially in cells with low mitotic activity, such as neurons and cardiomyocytes, to maintain cellular function [27,28]. Under certain pathological conditions, SUMOylation-regulated signaling pathways related to cardiovascular development and function are changed [29,30]. SUMOylation affects the function of cardiovascular proteins, emerging as key factors of various CVD progression [29]. In recent years, numerous new SUMOylation targets have emerged, and the important roles of SUMOylation in CVD progression have been continuously discovered [31,32].

## 2. SUMO Machinery and CVD

### 2.1. UBC9

**Figure 2:**
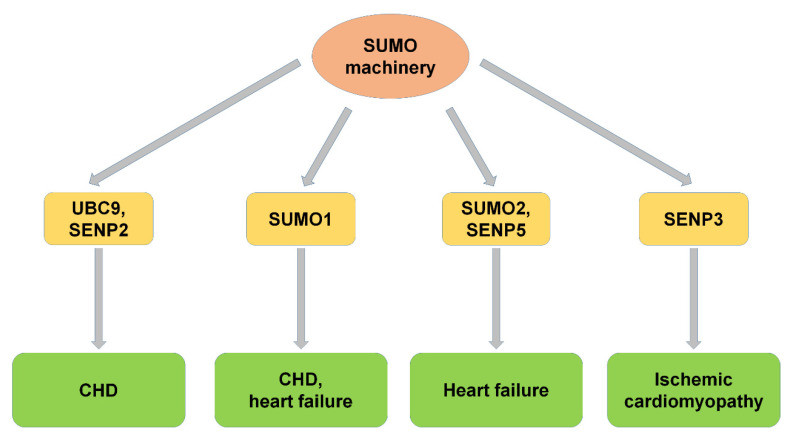
SUMO machinery and CVD. SUMO machinery, including UBC9, SUMO1, SUMO2, SENP2, SENP3, and SENP5, participated in the progression of CVDs, including congenital heart disease, heart failure, and ischemic cardiomyopathy. CHD: congenital heart disease.

UBC9 is the only E2 enzyme responsible for the function of GATA4 in cardiac development and function. UBC9 enhanced the transcriptional activity and altered the nuclear localization of GATA4 [33]. UBC9-mediated SUMOylation of GATA4 on lysine residue 366 (K366) promoted specific gene expression in cardiac pluripotent cells. Knockout of UBC9 in mice stimulated early embryonic death at embryonic day 3.5 [34]. UBC9-mediated SUMOylation induced a higher level of autophagy and increased autophagy flux and the incidence of cardiac development [35]. These studies indicated an important role of UBC9 in cardiac development (Figure 2).

### 2.2. SUMO1

The SUMOylation was mediated by the SUMO family, and SUMO1 was one of the most important members. SUMO1 plays a critical role in early heart development. Mice with SUMO1 knockout showed the CHD phenotype. Heterozygous and homozygous mice with higher ASD and VSD mortality due to SUMO1 knockout were rescued by cardiac expression of SUMO1 [36]. Thus, SUMO1 played a key role in normal cardiac development, and decreased SUMOylation activity induces cardiac pathology. The changed SUMOylation levels affect a variety of transcription factors and enzymes that maintain normal cardiac function.

SUMO1 was also found to improve the oxidative stress response during increased cardiac hypertrophy [37]. SUMO1 transfection with adenovirus after TAC, a model of heart failure induced by stress overload, prevented the occurrence of cardiac hypertrophy, blocked the negative effect of hydrogen peroxide on SERCA2a, and decreased the oxidative stress index in mice [38]. These studies indicate that SUMO1 plays important roles in normal cardiac function and suggest that SUMO1 prevents heart failure in a toxic environment (Table 1).

### 2.3. SUMO2

In previous studies, little is known about whether and how SUMO2/3 functions in CVD. It was reported that endogenous SUMO2 was important in maintaining normal endothelium-dependent vascular function [40], and SUMO2 upregulation disrupts the balance of the vascular environment and leads to endothelial dysfunction due to cholesterolemia [41]. Increased SUMO2 binding protein was found in the heart of SUMO2 transgenic mice, which showed the cardiomyopathy phenotype [41]. These results indicated that SUMO2-mediated SUMOylation maintains a great balance in normal conditions, and the imbalance of SUMOylation leads to certain pathological conditions, including heart failure.

### 2.4. SENP2

Studies have shown that SENP2 is associated with CHD, cardiac dysfunction, and embryonic development and is a key regulatory factor in early cardiac morphosis [13]. In transgenic mice with cardiac-specific SENP2 expression, overexpressed SENP2 leads to ASDs and VSDs, reduced proliferation of cardiomyocytes, and induced cardiomyopathy with aging [13]. SENP2 deconjugated SUMO from many transcriptional factors that played important roles in cardiac development [36]. SENP2 inhibits the transcriptional activity of NKX2-5 by promoting the uncoupling of SUMO1, affecting cardiac development and function [44]. These results suggested a crucial role of SENP2 in the pathogenesis of CHD.

### 2.5. SENP3

Interruption of blood supply to the heart is one of the leading causes of disability and death. However, little is known about the molecular events during cardiac ischemia [39] and how these changes function during cell death after reperfusion [45]. SUMOylation of proteins protects cells from several stresses, including ischemia-reperfusion. SENP3 has been reported to be an important determinant factor of cell survival after ischemic infarction [42]. It was found that ischemia-reperfusion significantly reduced the cytoplasmic level of SENP3 and accelerated cell death. It was suggested that SENP3-mediated deSUMOylation plays important roles in regulating myocardial cell survival after ischemic injury.

### 2.6. SENP5

SENP5 exhibits both endopeptidase and isopeptidase activities [46] and plays important roles in cell proliferation and survival [47,48]. Studies have shown that the level of SENP5, which mainly deconjugates SUMO2/3, is elevated in heart failure [43]. In mitochondria of the normal heart, SUMO1 modified Drp1 to increase its stability [47]. However, in a failing heart, the SENP5 level was increased. The increase of mitochondrial division mediated by Drp1 leads to changes in the morphology and structure of cardiac mitochondria, resulting in mitochondrial dysfunction, thereby inhibiting the proliferation of cardiomyocytes and aggravating apoptosis [43]. These results indicated that SENP5 plays important roles in heart failure and is a potential target for the treatment of heart failure.

## 3. SUMOylation of Proteins and CVD

### 3.1. Atherosclerosis

**Figure 3:**
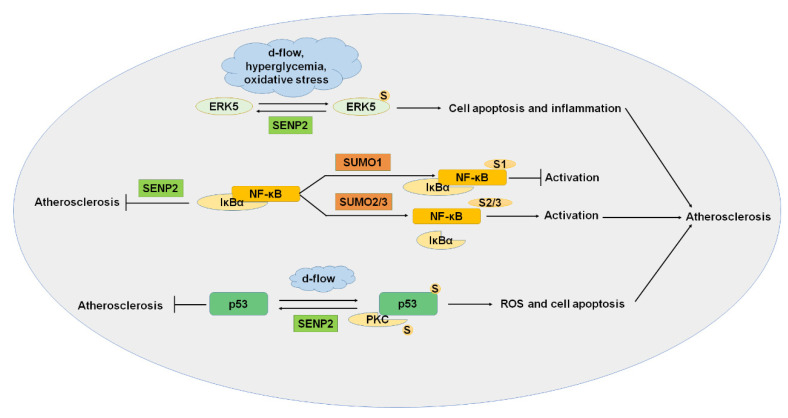
SUMOylation and atherosclerosis. ERK5, NF-κB, *p53*, and PKC were SUMOylated, and SUMOylation of these proteins played important roles in the progression of atherosclerosis. ERK5, extracellular signal-regulated kinase 5; PKC, protein kinase C. S: SUMO.

Atherosclerosis is a progressive inflammatory cardiovascular disease characterized by lipid plaque formation in the artery [2,49]. Endothelial cells form the main barrier to vascular permeability, preventing the formation of atherosclerosis [50]. Dysfunction of endothelial cells increases vascular wall permeability, lipid accumulation, and immune cell infiltration in large- and medium-sized artery walls, and subendothelial LDL (low-density lipoprotein) accumulation increases the rate of plaque formation [51,52]. Increasing evidence has shown that SUMOylation plays an important role in regulating inflammation, migration, actin filament remodeling, and apoptosis during the formation of atherosclerosis (Figure 3).

#### 3.1.1. ERK5 (Extracellular Signal-Regulated Kinase 5)

MAPK (mitogen-activated protein kinases) is a family of serine/threonine kinase composed of four major subfamilies that regulate cellular activities, including cell differentiation, proliferation, and apoptosis. ERK5 (or MAPK7) is one of the most important members of MAPK. ERK5 is not SUMOylated in normal conditions. However, the SUMOylation of ERK5 was induced by d-flow, hyperglycemia, and oxidative stress, and ERK5 SUMOylation induced cell apoptosis and inflammation. SENP2 protects endothelial cells by deSUMOylation of ERK5 to inhibit the pathological change [3]. It has shown that ERK5 activation plays an anti-inflammatory role in the progression of atherosclerosis [53]. It was reported that SUMOylation of ERK5 induced by H_2_O_2_ significantly inhibited the transcriptional activity of ERK5, causing inflammation in the onset and promoting the progression of atherosclerosis [54]. These results suggest that the SUMOylation of ERK5 is one of the most meaningful causes of atherosclerosis, and maintaining the balance of ERK5 SUMOylation is a potential treatment for atherosclerosis.

#### 3.1.2. NF-κB

NF-κB is a nuclear transcription factor and regulates cytokines and adhesion molecules released by leukocyte accumulation [55]. NF-κB in the cytoplasm binds to the inhibitor of κB (IκB) during the resting state. It was reported that endothelium-specific knockout of NEMO (NF-κB essential modulator) or dominant-negative IκBα inhibits atherosclerotic plaque formation [56]. NF-κB was SUMOylated, and SUMOylation of NF-κB plays a critical role in the pathogenesis of atherosclerosis. SUMO1-mediated SUMOylation of NF-κB inhibits ubiquitination-mediated degradation of IκBα and downregulates NF-κB activation [57]. The modification by SUMO2/3 promoted the detachment of IκBα from NF-κB and upregulated NF-κB activation, ultimately inducing atherosclerosis [57,58]. On the other hand, SENP2 negatively regulates inflammation through deSUMOylation of NEMO induced by genotoxic stress and ultimately inhibits atherosclerosis [58]. These results indicated that NF-κB plays important roles in atherosclerosis, and the regulation of NF-κB SUMOylation has become a potential therapeutic target for atherosclerosis.

#### 3.1.3. *p53*

*p53* is an important tumor suppressor gene that determines cell response to stress conditions, such as apoptosis and cell cycle arrest [59]. When cells are stimulated, nuclear *p53* is increased, and cell cycle regulators and pro-apoptotic genes are upregulated. The role of *p53* in atherosclerotic endothelial cells is complicated [60]. In d-flow, PKC mediates the SUMOylation of *p53* and induces reactive oxygen species (ROS), leading to the transfer of *p53* from the nucleus to the cytoplasm of endothelial cells. Combined with the anti-apoptotic factor Bax and Bcl-2, SUMOylated *p53* inhibited the anti-apoptotic and led to apoptosis, ultimately causing the occurrence of atherosclerosis [59]. On the other hand, SENP2 deSUMOylated *p53* and inhibited atherosclerosis [3]. p90 ribosomal S6 kinase (p90RSK) is a serine/threonine kinase with two kinase functional domains. D-flow activation of p90RSK resulted in the extranuclear output of SENP2 and increasing SUMOylation of *p53* and ERK5 [61]. These results indicated that deSUMOylation of *p53* by SENP2 may be an effective strategy to prevent atherosclerosis.

#### 3.1.4. PKC (Protein Kinase C)

PKC isoforms consist of a series of kinases chronically activated by hyperglycemia in diabetic patients and are essential in cell contractility and permeability. PKC is critical in regulating insulin resistance, gluconeogenesis, liver lipid metabolism, and key components of vascular homeostasis and is necessary for atherosclerosis. Changes in the PKC signaling pathway in diabetic patients may lead to various pathological results, thus accelerating DM-mediated atherosclerosis. PKC gene knockout reduced the size of plaques and reduced the production of inflammatory factors [62]. PKC was modified by SUMO1 at K465 and was deSUMOylated by SENP1 [62]. SUMOylation inhibited the function of PKC and played critical roles in the progression of atherosclerosis. The regulation of PKC SUMOylation may become a potential therapeutic target for DM-mediated atherosclerosis.

### 3.2. CHD (Congenital Heart Disease)

**Figure 4:**
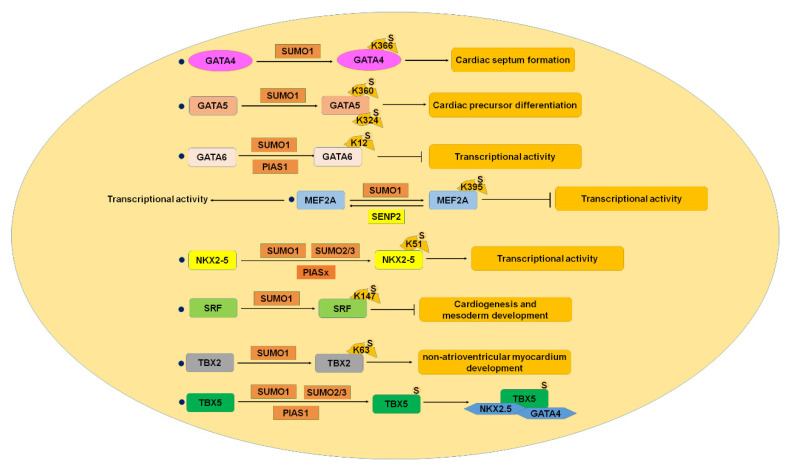
SUMOylation and CHD. GATA4-6, MEF2, NKX2-5, SRF, TBX2, and TBX5 were SUMOylated, and SUMOylation of these proteins played critical roles in cardiac development, whereas imbalance of SUMOylation caused congenital heart disease. CHD, congenital heart disease; MEF2, myocyte enhancer factor 2; SRF, serum responsive factor. S: SUMO.

The development of the vertebrate heart begins at the later stage of fetal development and involves a series of complex morphogenetic events, which are strictly regulated by the specific expression of various genes (Table 2) [29]. CHD is a congenital cardiac structural abnormality, and it is a common congenital defect in infants, accounting for about 1% of all newborns [4]. ASDs and VSDs are the most common CHDs, which are characterized by an existing connection between the atria and ventricles [5]. The interaction between different kinds of proteins is the basis of normal cardiac development, and the imbalance of their expression regulated by SUMOylation may lead to changes in protein function and the occurrence of CVD (Figure 4).

#### 3.2.1. GATA4

GATA4 is a transcription factor and one of the most important members of the GATA superfamily of DNA-binding transcription factors, which is reported to be one of the primary causes of CHD [85]. The GATA family plays important roles in regulating cardiac early stage and end-stage development, proliferation and differentiation of cardiac progenitor cells, and cardiac-specific gene expression [86]. GATA4 plays a key role in early heart development as one of the early markers of cardiomyocytes in embryonic development. GATA4 was SUMOylated, and the SUMOylation of GATA4 results in enhanced transcriptional activity and altered nuclear localization [87]. SUMO1 modification of GATA4 on lysine 366 promoted specific gene expression in cardiac pluripotent cells. The SUMOylation of GATA4 may play a role in a complex that regulates a set of genes needed to form cardiac septum. GATA4 mutation was found to be associated with non-syndromic cardiac septal defect [63]. Multiple GATA4 mutations were also found in patients with ASDs and VSDs [88]. Histological analysis suggests that the SUMOylation of GATA4 is essential in the early development of the heart, indicating that the regulation of GATA4 SUMOylation is a potential therapeutic target for CHD treatment.

#### 3.2.2. GATA5

GATA5 was found to be an important transcriptional factor in zebrafish heart development. GATA5 is reported to be modified by SUMO1 at lysine 324 (K324) and lysine 360 (K360). The SUMOylation sites’ mutation affected the transcriptional activity of GATA5 but did not affect the subcellular localization. Studies have shown that K324R mutant GATA5 could rescue the damaged cardiac precursor differentiation, while K360R mutant GATA5 cannot. In addition, the SUMOylation of GATA5 could restore cardiac insufficiency caused by cardiac dysplasia [64]. These results suggest that SUMOylation of GATA5 plays important roles in cardiac development.

#### 3.2.3. GATA6

GATA6 is one of the most important members of the DNA binding transcription factor in the GATA superfamily and is an important factor in the regulation of cardiac development [86]. GATA6 was modified by SUMO1 at lysine 12 (K12), and the specific E3 ligase PIAS1 preferentially promoted the conjugation of SUMO1 to GATA6 via its RING finger domain [20,65]. The SUMOylation of GATA6 inhibited its transcriptional activity but did not affect the subcellular localization [65]. The SUMOylation provides a new regulated mechanism for the modification of GATA-6 activity in cardiac development.

#### 3.2.4. MEF2 (Myocyte Enhancer Factor 2)

The MEF2 family is transcription factor that regulates muscle-specific genes involved in cardiac development, including MEF2A, MEF2B, MEF2C, and MEF2D [66]. Reducing the activity of MEF2 inhibits cardiomyocyte differentiation and cardiac development [71]. All these four transcription factors have the SUMO consensus sequence. Previous studies have shown that MEF2A, MEF2C, and MEF2D were modified by SUMO [89]. MEF2A can be modified by SUMO1 in vivo and in vitro. The SUMOylation of MEF2A inhibited its transcriptional activity, but the mutation of lysine 395 to arginine (K395A) in MEF2A enhanced the transcriptional activity of MEF2A [66]. SENP2 enhanced the transcriptional activity of MEF2A in activity dependent-regulation of MEF2A deSUMOylation. Taken together, these results showed that MEF2 played critical roles in embryonic cardiac development.

#### 3.2.5. NKX2-5

NKX2-5 is a cardiac-specific gene that regulates cardiac progenitor cell differentiation, mature and atrioventricular conduction system, and the formation of the atrioventricular outflow [90]. NKX2-5 was SUMOylated by SUMO1 on lysine 51, and SUMO E3 ligase PIASx enhanced the attachment between them [44]. NKX2-5 is modified by SUMO1 and can be modified by SUMO2/3 but to a much lesser degree than SUMO1, and SUMOylation promoted the transcription activity of NKX2-5 [91,92]. SUMOylation regulates NKX2-5 function by changing the interaction between NKX2-5 and related proteins and promotes stable complexes containing NKX2-5. The SUMOylation of NKX2-5 is necessary for normal cardiac development, whereas NKX2-5 mutation was associated with forming a variety of CHD [92]. These results indicate that NKX2-5 SUMOylation plays an important role in CHD and becomes a therapeutic target for CHD.

#### 3.2.6. SRF (Serum Responsive Factor)

SRF is a DNA binding protein family known as MADS Box, which has a highly conserved DNA binding/dimerization [93]. In vertebrates, SRF is mainly expressed in mesoderm and neuroectoderm tissues. SRF plays an important role during cardiac development, particularly in cardiogenesis and mesoderm development [94]. Serum stimulation activates SRF-mediated early gene transcription through various signal transduction pathways, such as newborn heart and smooth muscle-related genes [95]. SRF deficiency decreased cell proliferation and survival rate and increased cell apoptosis [96]. Studies have shown that SRF was SUMOylated by SUMO1 on its lysine residue 147 (K147), and the SUMOylation of SRF enhanced activation of the c-fos promoter but inhibited cardiac α-actin promoter [95]. These results suggest that the regulation of SRF SUMOylation plays critical roles in cardiac development.

#### 3.2.7. TBX2

TBX2 is a member of the T-Box transcription factors family and plays vital roles in cardiac cushion development [97]. Changes in TBX2 activity are associated with a variety of human congenital diseases and cancers. Changes in numerous factors associated with CHD are accompanied by changes in TBX2 [98]. It was reported that common genetic variation in the TBX2 promoter region is strongly associated with the cardiac development and occurrence of CHD [99]. TBX2 is associated with the non-atrioventricular myocardium in the atrioventricular and outflow canals during cardiac development. TBX2 was identified to be modified by SUMO1, and TBX2 SUMOylation plays an important role in cardiac development, especially non-atrioventricular myocardium in the atrioventricular and outflow canals [100]. Therefore, the SUMOylation of TBX2 plays an important role in cardiac development.

#### 3.2.8. TBX5

TBX5 is a member of the T-Box transcription factor family and plays a critical role in cardiac development [101]. In the early development of the heart, TBX5 acts as an upstream transcriptional activator to promote cardiomyocyte maturation [102]. In the late stage of heart development, TBX5 acts on the heart conduction system and maintains the function of mature cardiomyocytes [70,103]. TBX5 was SUMOylated by SUMO1, and SUMOylation enhanced the transcriptional activity of TBX5 [104]. The E3 ligase PIAS1 specifically enhanced the SUMOylation of TBX5 and enhanced the coordinate regulation with NKX2-5 and GATA4 [104]. These studies suggest the important roles of TBX5 in the progression of cardiac development.

### 3.3. HF (Heart Failure)

**Figure 5:**
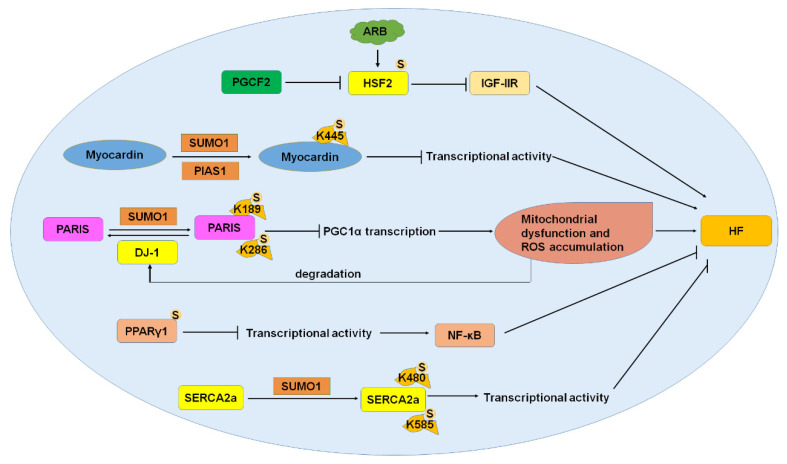
SUMOylation and HF. HSF2, myocardin, PARIS, PPARγ1, and SERCA2a were SUMOylated, and SUMOylation of these proteins played crucial roles in the progression of heart failure. HF, heart failure; HSF2, heat shock transcription factor 2; PARIS, parkin-interacting substrate; PPARγ1, peroxisome proliferation activated receptor γ1; SERCA2a, sarcoplasmic/endoplasmic reticulum Ca^2+^-ATPase 2a. S: SUMO.

HF is a complex disease caused by valve abnormality, coronary artery disease, hypertension, and other pathological changes [6]. Cardiac energy metabolism and mitochondrial biogenesis defects lead to HF and cardiac hypertrophy [7]. Cardiac hypertrophy is a transitional stage of HF that initiates a process of molecular and structural changes and lead to ventricular remodeling. Initially, persistent cardiac hypertrophy is an adaptive response and increases the risk of HF and sudden death [105]. Treatment with drugs for CVD is effective in reducing left ventricular hypertrophy and related mortality [106], and the SUMOylation pathway is involved in the development of HF and progression of cardiac hypertrophy (Figure 5).

#### 3.3.1. HSF2 (Heat Shock Transcription Factor 2)

Cardiac hypertrophy is a major feature of early hypertensive heart failure. Insulin-like growth factor II receptor (IGF-IIR) plays key roles in cardiomyocyte hypertrophy induced by hypertensive angiotensin II [72]. IGF-IIR is regulated by HSF2, which is modified by SUMO1 during HF [72]. PGCF2 (polycomb group ring finger 2) significantly reduced the SUMOylation of HSF2 in spontaneously hypertensive rat hearts. Inhibition of HSF2 SUMOylation induced cardiac hypertrophy in hypertensive rats. The treatment by angiotensin-receptor I blocker (ARB) restored the SUMOylation of HSF2 and alleviated cardiac defects in spontaneously hypertensive rats. The studies showed that the PGCF2-SUMO1-HSF2-IGF-IIR pathway had a profound effect on cardiac hypertrophy in patients with hypertensive HF [72]. These results suggested that the regulation of HSF2 SUMOylation is a potential target for the treatment of cardiac hypertrophy.

#### 3.3.2. Myocardin

Myocardin belongs to the SAP superfamily and is only expressed in cardiac smooth muscle during embryonic development and plays a key role in cardiomyocyte hypertrophy [107]. Myocardin affects the growth of cardiomyocytes by regulating the stability and metabolic activity of cardiomyocytes [108]. Myocardin promotes smooth muscle cell differentiation with a coactivator of SRF [109]. Loss of function of myocardin leads to serious defects in vascular smooth muscle development [110]. Abnormal expression of myocardin leads to hypertrophic cardiomyopathy and HF, and its mutation may lead to cardiovascular abnormalities in humans. It was reported that myocardin was SUMOylated by SUMO1 in K445, and SUMOylation of myocardin by SUMO1 and E3 PIAS1 reversed the expression of myocardial genes and enhanced the cardiac hypertrophy of primary neonatal cardiomyocytes [74]. These results indicated the critical roles of myocardin SUMOylation in the development of cardiac hypertrophy.

#### 3.3.3. PARIS (Parkin-Interacting Substrate)

PARIS is regulated by the ubiquitin proteasome system via the E3 ubiquitin ligase parkin [111]. PARIS is a transcriptional repressor containing four zinc finger domains and one KRAB domain to regulate DNA binding in the promoter region of target genes and play transcriptional repressor activity [112]. PARIS was SUMOylated by SUMO1 in K189 and K286 and was deSUMOylated by DJ-1 [75,76]. In cardiac hypertrophy, PARIS was SUMOylated and inhibited PGC1α transcription. As one of the major regulators of mitochondrial function and biogenesis, the PGC1α transcription decrease causes mitochondrial dysfunction and ROS accumulation [113]. The accumulation of ROS in cardiac hypertrophy induced the degradation of DJ-1 and augmented the SUMOylation of PARIS, and reduced the promoter activity of PGC1α [114]. These results indicated the critical roles of PARIS SUMOylation in the regulation of mitochondrial function, and inhibition of the SUMOylation of PARIS may be a potential treatment of cardiac hypertrophy.

#### 3.3.4. PPARγ1 (Peroxisome Proliferation-Activated Receptor γ1)

The utilization of cardiac energy is partially determined by the PPAR transcription factors, including PPARα, PPARβ, and PPARγ, and the heterodimer-forming RXRα as well as coactivator PGC-1α and inhibitor NCoR (nuclear receptor corepressor). The PPAR family regulates the transcription of genes related to energy regulation and lipid metabolism [115]. PPARγ1 plays critical roles in the development of myocardial fibrosis and the protection of cardiac oxidative stress. PPARγ1 was covalently modified by SUMO1 in K107 and K365 with E3 ligase PIAS1 and PIASxβ, and SUMOylation inhibited the transcriptional activity of PPARγ1 [77,116]. The SUMOylation of PPARγ1 regulated NF-κB in response to hypertrophy and inflammatory stimuli, suggesting a protective role of PPARγ1 SUMOylation in cardiac hypertrophy [115]. These results indicated that the SUMOylation of PPARγ1 is a potential target for the treatment of cardiac hypertrophy.

#### 3.3.5. SERCA2a (Sarcoplasmic/Endoplasmic Reticulum Ca^2+^-ATPase 2a)

SERCA2a is a key pump that regulates the calcium cycle in cardiomyocytes and is responsible for the reuptake of calcium during excitation-contraction coupling [117]. Reduced activity and expression of SERCA2a are markers of HF in the body [118,119]. It has been demonstrated that SERCA2a can be modified by SUMO1 at lysine 480 and 585, and SUMOylation is essential to maintain the stability and activity of SERCA2a [78]. The content of SUMO1 and SUMOylated SERCA2a was greatly reduced in heart failure. After the knockout of SUMO1, the activity of SERCA2a was also reduced, which accelerated the impairment of heart function and the death of animals [120]. After restoring SUMO1 levels, SERCA2a levels rose, and the heart function of mice and swine went into remission [121,122]. SUMOylation of SERCA2a increased the stability and activity of SERCA2a, and the contractility of the myocardium increased. The SUMOylation of SERCA2a is of great significance for the treatment of HF [123]. There is a small molecule, N106, that can activate E1 ligase and trigger the SUMOylation of SERCA2a and enhance the contractility of cardiomyocytes and improve ventricular function in heart failure mice [124]. These studies indicate that the SUMOylation of SERCA2a is a potential target and a promising treatment for HF.

### 3.4. Ischemic Cardiomyopathy

**Figure 6:**
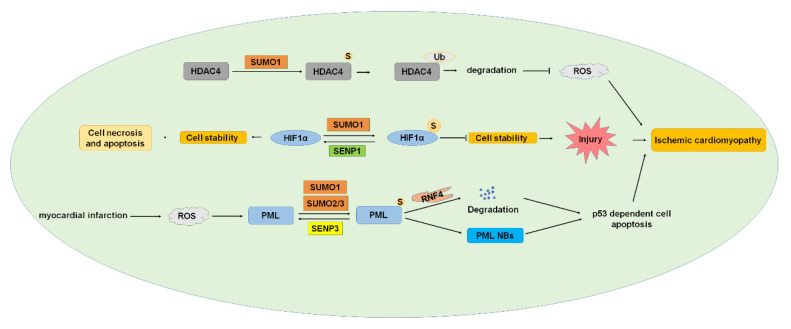
SUMOylation and ischemic cardiomyopathy. HDAC4, HIF1α, and PML were SUMOylated, and SUMOylation of these proteins played essential roles in developing ischemic cardiomyopathy. HDAC4, histone deacetylase 4; HIF1α, hypoxia-inducible factor 1α; PML, promyelocytic leukemia. S: SUMO.

AMI (acute myocardial infarction) is ischemic heart disease with acute hypoxia in the myocardial tissue and is one of the leading causes of death [125]. Restoration of reperfusion is the main therapeutic method to limit the size of myocardial infarction, save the viable myocardium, maintain the systolic function of the heart, and delay the occurrence of HF [14,82]. Readjustment of blood flow restores oxygen and nutrient supply but may damage the cardiac muscle and induce myocardial I/R injury. Studies of animal models of AMI have shown that fatal reperfusion injury accounts for half of the myocardial infarction [126]. However, the underlying mechanisms of myocardial I/R injury have not been fully elucidated [127]. SUMOylation has been found to play a significant role in myocardial I/R injury, and several SUMOylated proteins interfere with multiple mechanisms of myocardial I/R injury (Figure 6).

#### 3.4.1. HDAC4 (Histone Deacetylase 4)

HDAC induces deacetylation, chromatin concentration, and transcription inhibition [128]. According to its function and sequence homology, HDAC can be divided into four types. HDAC4 belongs to class II HDACs and is distributed in the nucleus and cytoplasm. HDAC4 is highly expressed in skeletal muscle, brain, and heart [129,130,131]. HDAC4 contributes to myocardial I/R injury due to mitochondrial damage and reactive oxygen species (ROS) during reperfusion. Inhibiting HDAC4 expression promoted cardiac repair, improved cardiac function, and inhibited cardiac remodeling [132]. HDAC4 was modified by SUMO1, and SUMOylation of HDAC4 reduced the generation of ROS [80]. The SUMOylation of HDAC4 mediates ubiquitination and promotes the degradation of HDAC4 in I/R, which may be related to the decrease of ROS production after the degradation of HDAC4 [80]. ROS-mediated oxidative stress has been widely recognized as one of the important mechanisms of myocardial I/R injury [133]. These results indicate that the SUMOylation of HDAC4 plays important roles and is a potential therapeutic target for myocardial I/R injury.

#### 3.4.2. HIF1α (Hypoxia-Inducible Factor 1α)

HIF1α is an important transcription factor that plays a critical role in protecting the I/R process by participating in the transcription response under hypoxia conditions. HIF1α was modified by SUMO1 to regulate the expression of heme oxygenase-1 in the rostral ventrolateral medulla (RVLM), playing a critical role in the regulation of brain death [8]. SENP1-mediated deSUMOylation of HIF1α leads to cell necrosis and apoptosis and plays an indispensable role in myocardial infarction after I/R in response to cell hypoxia. SUMOylation of HIF1α reduces its stability and leads to degradation, whereas SENP1 deSUMOylates HIF1α and improves its stability [134,135]. These studies showed that the decrease of SENP1 levels aggravated the I/R injury of cardiomyocytes by the HIF1α-dependent pathway, and the overexpression of HIF1α reversed the worsening effect of SENP1 downregulation on cell death [82]. These results suggest that the SUMOylation of HIF1α plays an important role in I/R injury and might be a potential target of AMI treatment.

#### 3.4.3. PML (Promyelocytic Leukemia)

PML is the important organizer of nuclear bodies (NBs) and is expressed in several isoforms, including PML I to VII [136]. PML is SUMOylated by SUMO1 and SUMO2/3 and de-SUMOylated by SENP3 [42,137], and SUMOylation is critical in PML NBs formation [83]. Human ring-finger protein 4 (RNF4) is an E3 ubiquitin ligase and targets poly-SUMOylated proteins to degrade by the ubiquitin-proteasome pathway [138]. RNF4 is involved in controlling the degradation of PML and the transcriptional activity of several transcription factors [139,140]. PML is an ROS sensor, and myocardial infarction (MI) generated ROS, and ROS induced the increase of PML SUMOylation and PML-NB formation, leading to p53-dependent cell apoptosis. Overexpression of PML or knockdown of RNF4 enhances p53 recruitment and activation of PML SUMOylation and PML NBs accumulation, which aggravated ischemia-induced cardiomyocyte apoptosis in vivo and cell injury [84]. These results indicated the important roles of PML SUMOylation in ischemia and suggested that intervening in the dynamic pattern of PML SUMOylation under oxidative stress is a potential treatment of MI.

#### 3.4.4. Target Protein Network

It has been reported that SUMOylation is enhanced to protect the brain during cardiac ischemia [141,142]. To identify the potential target proteins of SUMOylation in the mouse heart with cardiac I/R, an unbiased proteomic analysis was conducted. There were 102 proteins that significantly interacted with SUMO1, including the composition of the SUMO mechanism and the ubiquitin proteasome system [143,144], multiple subunits of 20S proteasome core particle, and metabolic enzymes involved in glycolytic and oxidative metabolic pathways [145]. High molecular weight SUMO1 conjugates were significantly reduced 24 h after reperfusion. In total, 15 proteins showed significant changes in SUMOylation at ischemia, and 13 and 12 proteins showed changes in SUMOylation at 1 and 24 h after reperfusion, respectively. Further, 55 proteins were found to be potential SUMO2/3 target proteins, including ubiquitin and proteasome subunits, gene expression regulators and transcription factors, RNA binding proteins and splicing regulators, heat shock proteins, and specific proteins that play important roles in cardiac function. Myocardial ischemia decreased the SUMO level of the whole protein and the number and catalytic activity of de-SUMO enzymes [145]. The heart SUMO2 target protein undergoes a dynamic adaptation process in cardiac I/R. Only one protein was SUMOylated differently at ischemia, and 8 and 11 target proteins were significantly altered 1 and 24 h after reperfusion, respectively. SENP3 is the major deSUMOylation enzyme in mouse hearts [145]. These results identified potential targets of SUMOylation during cardiac I/R and provided potential therapeutic targets for the treatment of cardiac ischemia.

## 4. Concluding Remarks and Perspective

Cardiovascular development and function are regulated by complex factors and signal pathways, and cardiovascular disorders lead to a series of stress responses [146,147]. SUMOylation is the PTM of proteins by a series of enzyme-linked reactions. Acute cardiac hypoxia induces elevated late sodium current and action potential prolongation in the heart and causes pro-arrhythmic change by SUMOylation of Nav1.5 channels [148]. In the decades since its discovery, studies related to SUMOylation in a variety of diseases have been extensively reported, including cardiovascular development and function [29,30].

**Figure 7:**
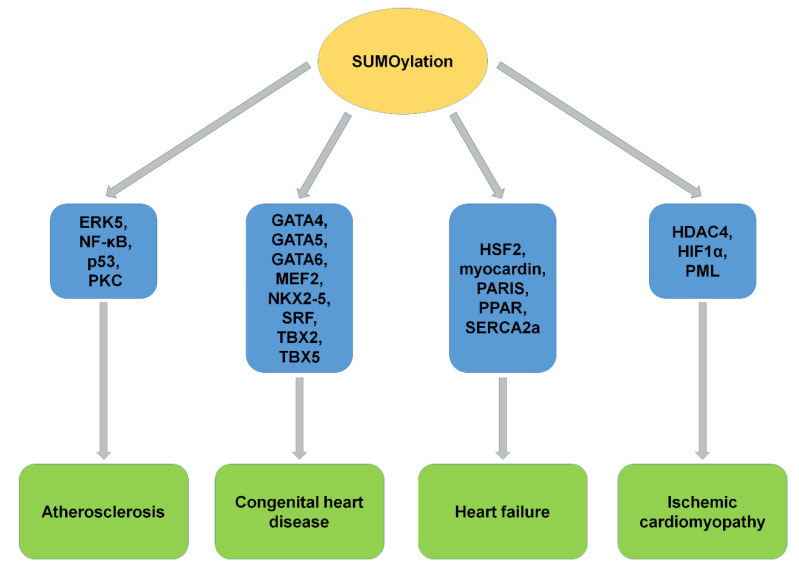
The SUMOylation of substrates and CVD. SUMOylation of target proteins played critical roles and showed potential clinical implications in CVD, including atherosclerosis, congenital heart disease, heart failure, and ischemic cardiomyopathy. ERK5, extracellular signal-regulated kinase 5; PKC, protein kinase C; MEF2, myocyte enhancer factor 2; SRF, serum responsive factor; HSF2, heat shock transcription factor 2; PARIS, parkin-interacting substrate; PPARγ1, peroxisome proliferation activated receptor γ1; SERCA2a, sarcoplasmic/endoplasmic reticulum Ca^2+^-ATPase 2a; HDAC4, histone deacetylase 4; HIF1α, hypoxia-inducible factor 1α; PML, promyelocytic leukemia.

Many factors regulated by SUMOylation and the imbalance of protein SUMOylation are involved in different types of CVDs, including atherosclerosis, CHD, HF, and ischemic cardiomyopathy. The previous studies indicated that SUMOylation is closely related to the occurrence and development of CVDs (Figure 7). The disruption of SUMOylation balance leads to severe CVDs. However, the molecular mechanism of SUMOylation in CVD has been preliminarily studied. It is possible to determine endogenous SUMOylated proteins under native conditions and identify endogenous SUMOylation sites on factors important in cardiac functions [149,150]. More SUMOylated proteins will be discovered during cardiovascular development and maintenance of cardiac function with further studies. Although the therapeutic strategies of CVDs have been greatly improved, there is still a lack of accurate, effective, and scientific unified treatment. Target therapy is one favorable way for future medical development and destination of CVD treatment. SUMOylation not only regulates the function of target proteins but also affects other post-translational modification processes. It is possible that SUMOylation co-regulates the same physiological process with other post-translational modifications. Therefore, the synergistic effects of SUMOylation and other post-translational modifications on the progression of CVD should be considered in the clinical treatment. Exploring the dynamic balance between the target of SUMOylation and also other PTM will help to better understand the biological functions of SUMOylation and the clinical treatment of CVDs.

## Figures and Tables

**Table 1:** SUMO machinery and CVD.

SUMO Machinery	Disease	Biophysical and Biological Effects of SUMOylation
UBC9	CHD	UBC9-mediated SUMOylation induced a higher level of autophagy and increased autophagy flux and the incidence of cardiac development [35,39].
SUMO1	CHD and HF	SUMO1 plays a key role in normal cardiac development and decreased SUMOylation activity induces cardiac pathology [36].
SUMO2	HF	Endogenous SUMO2 is important in maintaining normal endothelium-dependent vascular function [40], and SUMO2 upregulation leads to endothelial dysfunction due to cholesterolemia [41].
SENP2	CHD	Overexpression of SENP2 led to ASD and VSD, reduced proliferation of cardiomyocytes, and induced cardiomyopathy with aging [13].
SENP3	Ischemic cardiomyopathy	SENP3-mediated deSUMOylation plays an important role in the regulation of myocardial cell survival after ischemic injury [42].
SENP5	HF	SENP5 level increased in the failing heart, and the increase of mitochondrial division mediated by Drp1 leads to changes in the morphology and structure of cardiac mitochondria, resulting in mitochondrial dysfunction [43].

CHD: congenital heart disease; HF: heart failure.

**Table 2:** The roles of SUMOylation of substrates in CVD.

Disease	Protein Substrate	Biophysical Function	Biophysical and Biological Effects of SUMOylation
Atherosclerosis	ERK5	ERK5 plays anti-inflammatory role in regulating PPARγ and KLF2 and inhibiting TNFα mediated adhesion gene expression.	SUMOylation of ERK5 significantly inhibits the transcriptional activity of MEF2, causing inflammation in the onset and promoting the progression of atherosclerosis [32].
	NF-κB	NF-κB is a nuclear transcription factor and is one of the earliest known cardiac progenitor cell markers.	SUMO1-mediated SUMOylation of NF-κB downregulates its activation, whereas SUMO2/3 leads to the detachment of NF-κB from IκBα and activation [57,58].
	*p53*	*p53* is an important tumor suppressor gene that regulates cell apoptosis and cell cycle.	SUMOylated *p53* promotes cell apoptosis and causes the occurrence of atherosclerosis, whereas deSUMOylated *p53* inhibits atherosclerosis [59].
	PKC	PKC plays an important role in cell growth, apoptosis, inflammation, and contractility of cardiomyocytes.	SUMOylation of PKC inhibits the kinase activity of PKC and accelerates the occurrence of atherosclerosis [4].
CHD	GATA4	GATA4 is one of the most important members of the GATA superfamily and is known to be the primary cause of CHD.	SUMOylation of GATA4 promotes cardiac specific gene expression in pluripotent cells and regulates the development of CHD [63].
	GATA5	GATA5 is an important transcription factor in cardiac development.	SUMOylation of GATA5 restores cardiac insufficiency caused by cardiac dysplasia and plays an important role in cardiac development [64].
	GATA6	GATA6 is one of the most important members of the GATA superfamily and is an important factor in the regulation of cardiac development.	SUMOylation of GATA6 inhibits its transcriptional activity in cardiac development [65].
	MEF2	The MEF2 family contains transcription factors that regulate the activity of muscle-specific genes and involved in cardiac development.	MEF2A, MEF2C, and MEF2D were modified by SUMO, and SUMOylation affects their transcriptional activity [66].
	NKX2-5	NKX2-5 is involved in the differentiation of cardiac progenitor cells and the formation of atrioventricular septum and atrioventricular outflow tract.	SUMOylation promotes the transcriptional activity of NKX2-5 and is associated with the progression of CHD [67].
	SRF	SRF is a member of the DNA binding protein family MADS Box and is involved in cardiac development.	SUMOylation of SRF enhanced activation of c-fos promoter, but inhibited cardiac α-actin promoter, playing critical roles in cardiac development [68].
	TBX2	TBX2 is a T-Box transcription factor and plays vital roles in cardiac cushion development.	TBX2 SUMOylation plays an important role in cardiac development, especially non-atrioventricular myocardium in the atrioventricular and outflow canals [69].
	TBX5	TBX5 is a T-Box transcription factor and plays a critical role in cardiac development.	SUMOylation of TBX5 enhanced the transcriptional activity of TBX5 and enhanced the coordinate regulation with NKX2-5 and GATA4, regulating the progression of cardiac development [70,71].
HF	HSF2	HSF2 plays a key role in cardiomyocyte apoptosis and hypertrophy induced by hypertensive angiotensin II.	HSF2 is modified by SUMO1 during HF, and PGCF2 significantly reduced the SUMOylation of HSF2 in spontaneously hypertensive rat hearts [72,73].
	Myocardin	Myocardin regulates the growth of cardiomyocytes by regulating the metabolic activity and plays a key role in the process of cardiomyocyte hypertrophy.	SUMOylation enhanced the activity of myocardin and leads to an increase in cardiac-specific gene expression in cardiomyocytes and promoted myocardin-mediated cardiac hypertrophy [74].
	PARIS	PARIS regulates DNA binding in the promoter region of target genes and plays transcriptional repressor activity.	In cardiac hypertrophy, SUMOylated PARIS inhibited PGC1α transcription to regulate mitochondrial function [75,76].
	PPARγ1	PPARγ1 regulates the utilization of cardiac energy substrates.	SUMOylation of PPARγ1 inhibits the transcriptional activity and plays important roles in cardiac hypertrophy [77].
	SERCA2a	SERCA2a regulates the calcium cycle in cardiomyocytes and is responsible for the reuptake of calcium during excitation-contraction coupling.	SUMOylation is essential to maintain the stability and activity of SERCA2a [78,79].
Ischemic cardiomyopathy	HDAC4	HDAC4 is a histone deacetylase and is highly expressed in heart, brain, and skeletal muscle.	SUMOylation of HDAC4 mediates ubiquitination and promotes the degradation of HDAC4 in I/R and decreases the production of ROS [80,81].
	HIF1α	HIF1α is an important transcription factor that plays critical roles under hypoxia conditions.	HIF1α SUMOylation regulates the expression of heme oxygenase-1 in rostral ventrolateral medulla (RVLM), whereas its deSUMOylation leads to cell necrosis and apoptosis [82].
	PML	PML is the important organizer of NBs.	PML SUMOylation is critical in PML NBs formation [83] and aggravates ischemia-induced cardiomyocyte apoptosis in vivo [84].

ERK5, extracellular signal-regulated kinase 5; PKC, protein kinase C; CHD, congenital heart disease; MEF2, myocyte enhancer factor 2; SRF, serum responsive factor; HF, heart failure; HSF2, heat shock transcription factor 2; PARIS, par-kin-interacting substrate; PPARγ1, peroxisome proliferation activated receptor γ1; SERCA2a, sarcoplas-mic/endoplasmic reticulum Ca2+-ATPase 2a; HDAC4, histone deacetylase 4; HIF1α, hypoxia-inducible factor 1α; PML, promyelocytic leukemia.
